# New Insight into Antimicrobial Compounds from Food and Marine-sourced *Carnobacterium* Species through Phenotype and Genome Analyses

**DOI:** 10.3390/microorganisms8071093

**Published:** 2020-07-21

**Authors:** Simon Begrem, Flora Ivaniuk, Frédérique Gigout-Chevalier, Laetitia Kolypczuk, Sandrine Bonnetot, Françoise Leroi, Olivier Grovel, Christine Delbarre-Ladrat, Delphine Passerini

**Affiliations:** 1 University of Nantes, 44035 Nantes Cedex 1, France; simon.begrem@etu.univ-nantes.fr (S.B.); olivier.grovel@univ-nantes.fr (O.G.); 2 IFREMER, BRM, EM3B Laboratory, 44300 Nantes Cedex 3, France; flora.ivaniuk@free.fr (F.I.); frederique.gigout@ifremer.fr (F.G.-C.); Laetitia.Kolypczuk@ifremer.fr (L.K.); Sandrine.Bonnetot@ifremer.fr (S.B.); Francoise.Leroi@ifremer.fr (F.L.); Christine.Delbarre.Ladrat@ifremer.fr (C.D.-L.)

**Keywords:** lactic acid bacteria, antimicrobial activity, *Carnobacterium* spp., hydrogen peroxide, bacteriocin, RiPP, NRPS, terpene, natural product, genome mining

## Abstract

*Carnobacteriummaltaromaticum* and *Carnobacterium* *divergens*, isolated from food products, are lactic acid bacteria known to produce active and efficient bacteriocins. Other species, particularly those originating from marine sources, are less studied. The aim of the study is to select promising strains with antimicrobial potential by combining genomic and phenotypic approaches on large datasets comprising 12 *Carnobacterium* species. The biosynthetic gene cluster (BGCs) diversity of 39 publicly available *Carnobacterium* spp. genomes revealed 67 BGCs, distributed according to the species and ecological niches. From zero to six BGCs were predicted per strain and classified into four classes: terpene, NRPS (non-ribosomal peptide synthetase), NRPS-PKS (hybrid non-ribosomal peptide synthetase-polyketide synthase), RiPP (ribosomally synthesized and post-translationally modified peptide). In parallel, the antimicrobial activity of 260 strains from seafood products was evaluated. Among the 60% of active strains, three genomes were sequenced and submitted to a dereplication process. *C. inhibens* MIP2551 produced a high amountof H_2_O_2_, probably thanks to the presence of four oxidase-encoding genes. *C. maltaromaticum* EBP3019 and SF668 strains were highly efficient against *Listeria monocytogenes*. A new extracellular 16 kDa unmodified bacteriocin in the EBP3019 strain and five different bacteriocins in SF668 were highlighted. In this study, the overview of antimicrobial BGC and inhibitory activities of *Carnobacterium* spp. allowed the prediction of potential innovative natural products that could be relevant for biotechnological applications.

## 1. Introduction

Lactic acid bacteria (LAB) are microaerophilic Gram-positive bacteria capable of fermenting sugars into lactic acid. Due to this feature, LAB are involved in various food fermentations such as dairy, meat, or vegetable products. Moreover, they are ubiquitous microorganisms present in many terrestrial environments, ranging from soil, plants, or animals. LAB can also be found in marine environments such as coastal and estuarine sediment or in fish gastrointestinal tract [1]. LAB have developed strategies to outcompete microorganisms in these various ecosystems, such as nutritional competition, environmental acidification, or production of secondary metabolites [2].

The production of active secondary metabolites by LAB is well documented. They include end or by-products such as organic acids, diacetyl, and hydrogen peroxide [3], and active peptides named bacteriocins [4,5,6]. These peptides exhibit antimicrobial activities against Gram-positive bacteria and rarely against Gram-negative bacteria and fungi [7]. Bacteriocin production is strain-dependent, and gene clusters allowing the production of such bacteriocins seem to be subjected to evolutionary selection, favoring the survival of specific bacterial strains in their environment [8,9]. Various classifications of bacteriocins have been proposed [10,11,12]. In this paper, bacteriocins will be designated following the classification of ribosomally synthesized and post-translationally modified peptides (RiPPs) [13]. In contrast to RiPPs, only two active secondary metabolites biosynthesized by a hybrid non-ribosomal peptide synthetase/polyketide synthase (NRPS/PKS) were described in LAB. One of them is involved in oxidative stress resistance [14], and the other inhibits Gram-positive bacteria growth [15].

The production of secondary metabolites can be encoded by different genes co-localized in genetic clusters within the genome called biosynthetic gene clusters (BGCs). Genome mining is a computational analysis approach that allows in silico prediction of BGCs in genomes leading to the identification of novel natural products [16,17,18]. A wide diversity of natural products encoded by BGCs can, thus be predicted by this approach, e.g., terpenes, RiPP, PKS (synthesized by a polyketide synthase), NRPS (synthesized by a non-ribosomal peptide synthetase), and hybrid NRPS/PKS. The dereplication process consists of the comparison of these predictions with databases to identify original BGCs. Phylogenetically-related organisms isolated from different ecological niches likely exhibit different genetic and phenotypic traits [19,20] and have the potential to biosynthesize various secondary metabolites [21]. In particular, organisms of marine origin are a promising source of innovative secondary metabolites [22,23].

*Carnobacterium* strains are lactic acid bacteria associated with food products or marine environments [24]. Twelve species have been reported: *C. alterfunditum*, *C. antarcticum*, *C. divergens*, *C. funditum*, *C. gallinarum*, *C. jeotgali*, *C. iners*, *C. inhibens*, *C. maltaromaticum*, *C. mobile*, *C. pleistocenium,* and *C. viridans*. Among them, *C. maltaromaticum* and *C. divergens* are frequently isolated from meat and fish products and are used for biopreservation applications [25]. They were shown to prevent Gram-positive bacteria from growing, such as pathogenic (e.g., *Listeria monocytogenes*) or food-spoiling bacteria, through the production of bacteriocins [24,26,27]. To date, 18 different bacteriocins have been described in *Carnobacterium* spp., and most of them were produced by *C. maltaromaticum* and *C. divergens* strains isolated from food products (Table S1). Among other species, some strains displayed activities against Gram-negative bacteria and fungi, although the mechanisms have not been identified yet [28,29]. Moreover, a hybrid NRPS/PKS BGC was predicted in *C. divergens* V41 strain, but no product or biological activity has been linked to this cluster yet [30].

The aim of this study was to investigate the antimicrobial activities and potential compounds produced by *Carnobacterium* spp. strains by two complementary approaches. First, the extent of BGCs diversity was evaluated from 89 *Carnobacterium* spp. publicly available genomes after validation of the species affiliation by phylogenomic analyses. Second, the antimicrobial activity of an unexplored collection of 260 *Carnobacterium* spp. isolates from seafood products was investigated. The genome of three atypical strains was sequenced and analyzed to highlight genomic specificities, using a dereplication process from genome mining. Besides the overview of BGCs distribution according to *Carnobacterium* species and their ecological niches, this study allowed the identification of new natural products potentially active.

## 2. Materials and Methods 

### 2.1. Carnobacterium spp. Public Genome Dataset

A total of 89 *Carnobacterium* spp. genomes were collected from the NCBI Assembly database (Table S2). Their 16S rDNA sequences were compared to identify the *Carnobacterium* species. Sequence alignment was performed with MUSCLE [31] and the maximum likelihood phylogenic tree was constructed using MEGA6 [32]. Phylogenetically closely related organisms, namely *Aerococcus urinaequii* CCUG 28094 (Culture Collection University of Gothenburg, Sweden), *Granulicatella adaciens* ATCC 49175 (American Type Culture Collection, USA), and *Pisciglobus halotolerans* DSM 27630 (German Collection of Microorganisms and cell cultures, Germany), were included in the phylogenetic analyses. *Lactococcus garvieae* ATCC 49156 was used as the outgroup. The OrthoANI-Usearch (OAU) software from EZbiocloud [33] was used to calculate the Average Nucleotide Identity (ANI) between the *Carnobacterium* spp. strains.

### 2.2. EBP3019, SF668, and MIP2551 Genome Sequencing and Automatic Annotation

One isolated colony was grown in 10 mL Brain Heart Infusion (BHI, Biokar Diagnostic, Beauvais, France) medium at 26 °C overnight. Five milliliters were centrifuged, and genomic DNA was extracted using the Wizard Genomic DNA Purification Kit (Promega, Madison, WI, USA). The protocol provided by the supplier was slightly modified with a supplementary prior lysis step as follows: 600 µL of Tris buffer containing 1 mM ethylenediaminetetraacetic acid (EDTA) and 10 mg/mL lysozyme were added and incubated for 1 h at 37 °C. The DNA was harvested in 25 µL of H_2_O and quantified with the NanoVue photometer (VWR, Fontenay-sous-Bois, France). The genomic DNA was sequenced by an Illumina Hi-Seq 2500 system with a read length of 2 × 250 bp (Genoscreen, Lille, France). A total of 1,164,664 reads for the MIP2551 strain, 807,963 for the SF668 strain, and 891,855 for the EBP3019 strain were produced. The coverage was over 50×. After quality filtering, *de novo* assembly was performed with SPAdes v3.8.0 [34]. A total of 42 contigs for the MIP2551 strain, 109 contigs for the EBP3019 strain, and 81 contigs for the SF668 strain were obtained, accounting for a total genomic length of 2.4 Mb, 3.4 Mb, and 3.4 Mb, respectively. The automatic annotation was carried out by the MicroScope platform [35].

### 2.3. BGC Prediction

All *Carnobacterium* spp. genomes were submitted to the RAST^nmpdr^ (Rapid Annotation using Subsystems technology) [36] and MicroScope platform for automatic annotation [35].

Genes encoding the main enzymes that contribute to H_2_O_2_ accumulation or recycling were identified from the automatic annotation—lactate oxidase (lox, EC.1.13.12.4), pyruvate oxidase (pox, EC.1.2.3.3) [37], α-glycerophosphate oxidase (glpO, EC.1.1.3.21) [38], and catalase (EC.1.11.1.6).

BGCs putatively involved in antimicrobial compound biosyntheses were identified by combining the AntiSMASH 5.0 [39] and BAGEL 4 [40] softwares. AntiSMASH is a web-based pipeline able to predict a large diversity of BGCs such as RiPPs, NRPS, PKS, terpenes, and siderophores. BAGEL is specialized in bacteriocin and RiPP cluster prediction. For each predicted BGC class, the presence of a minimal gene subset required for BGCs was investigated as follows. For RiPP clusters, the presence of a structural gene (core gene), an immunity protein-encoding gene, and accessory genes leading to post-translational modifications was investigated. When the core or immunity protein gene was not predicted, it was manually searched using Artemis [41] to predict small coding sequences (CDS). BLASTp was used to compare core sequences, conserved domains, and genetic environments of the *Carnobacterium* spp. BGCs. The hydrophobicity and high isoelectric point for the predicted head to tail cyclized peptide core sequence were checked according to Gabrielsen et al. with GPMAW_lite_ [42,43]. SignalP-5.0 was used to predict extracellular proteins and the cleavage position between the peptide signal and the core peptide [44]. For NRPS/PKS clusters identified by AntiSMASH, the presence of the required initiation and termination modules was checked.

A BGC network was built using the BiG-SCAPE software [45]. This network includes 77 BGCs previously predicted with AntiSMASH and BAGEL. When available, the genetically closest BGCs, already described in the literature were included. The network was built with a local mode and a cutoff parameter of 0.7. The layout of the network was done with BiG-SCAPE for spacing and connections between the different nodes and with Cytoscape for colorization and connector width based on the squared similarity parameter.

### 2.4. Carnobacterium spp. Strains, Growth Conditions, and Identification

For this study, 260 *Carnobacterium* spp. strains isolated by Ifremer (Nantes, France) from seafood products (smoked salmon, shrimps, and cod), and 12 reference strains were used (Table S3). Strains were stored at −80 °C in 15% (v/v) glycerol. Two successive pre-cultures were performed in BHI medium at 26 °C for 24 h.

Strains were identified using partial 16S rDNA sequencing. Briefly, 500 µL of culture suspensions were centrifuged for 20 min at 4500 *g*. Pellets were washed twice in 500 µL of 10 mM Tris containing 1 mM EDTA and resuspended in 200 µL of the same buffer before heating for 10 min at 95 °C. After centrifugation for 10 min at 4500× *g,* 1 µL of supernatant was used for PCR amplification of the 16S rRNA gene using the universal primers, E8-F (5′-AGAGTTTGATCATGGCTCAG-3′) and 1489R (5′-GTTACCTTGTTACGACTTCAC-3′). PCR amplification was performed under the following conditions: initial denaturation at 95 °C for 5 min; 30 cycles of amplification, including three steps (95 °C for 30 s, 52 °C for 30 s, 72 °C for 1 min), final extension for 10 min at 72 °C using a T100^TM^ Thermal Cycler (Bio-Rad, Hercules, CA, USA) in a final volume of 50 µL. The master mix 1X with the Dream*Taq* Green polymerase (Thermo Fisher Scientific, Waltham, MA, USA) was used. PCR products were purified, and the V1–V4 region was sequenced using the E8-F primer (Genoscreen, Lille, France).

### 2.5. Spot-on Lawn Assays

*Carnobacterium* spp. strains (260 from Ifremer collection and 12 references) were screened for antimicrobial activity by the spot-on lawn method adapted from Matamoros et al. [46]. Seventeen indicator strains involved in human infections, fish diseases, and food spoilage (Table S3) were used: *Enterococcus faecalis* CIP 105042 (Collection of the Institut Pasteur, France), *Staphylococcus epidermidis* CIP 68.21, *Listeria innocua* CIP 107775, *Listeria monocytogenes* RF191 [47], *Vagococcus salmoninarum* CIP 104684^T^, *Lactococcus garvieae* CIP 102507^T^, *Carnobacterium divergens* V41 [48], *Brochothrix thermosphacta* CIP 103251^T^, *Escherichia coli* JM109, *Vibrio parahaemolyticus* LMG 2850, *Pseudomonas fluorescens* CIP 69.13^T^, *Morganella morganii* CIP A231^T^, *Aeromonas salmonicida* CIP 103209^T^, *Vibrio harveyi* LMG 4044, *Chromobacterium violaceum* CIP 103350^T^, *Candida albicans* DSM 1386, and *Aspergillus fumigatus* MMS839 from the MMS laboratory fungal culture collection (Mer Molécules Santé Laboratory, Nantes University, France).

Ten microliters of 24-h culture broth or cell-free supernatants (CFSs) were spotted on the surface of the soft agar medium seeded with one of the bacterial or yeast indicator strains. Indicator bacterial strains were prepared as follows—two successive cultures were performed in 10 mL appropriate medium at 26 °C for 24 h. Except for *Aeromonas salmonicida*, *Chromobacterium violaceum*, and *Pseudomonas fluorescens*, which were grown in Tryptic Soy Broth (TSB, Biokar Diagnostic, Beauvais, France), all bacteria grew in BHI medium. *Candida albicans* was cultured in Lysogeny Broth (LB, 1% Tryptone; 0.5% Yeast extract; 1% NaCl). Bacterial indicator strains and *Candida albicans* were then inoculated in 20 mL of the culture medium supplemented with 0.5% agar to reach an initial concentration of 10^4^ CFU/mL and poured into one square Petri dish (120 × 120 mm). A 10^6^ spores/mL *Aspergillus fumigatus* MMS839 suspension was prepared by scraping cells with sterile water from a 96-h culture on Dextrose Casein Agar medium (DCA, Detroit, MI, USA). Three milliliters of spore suspension was left for 5 min on a 0.5% agar Mueller Hinton Broth (DCA, Detroit, MI, USA) surface in a square Petri dish (120 × 120 mm). The excess of liquid was discarded, and then the *Carnobacterium* spp. cultures were spotted. Clear haloes observed after 24 or 48-h incubation at 26 °C indicated growth inhibition.

### 2.6. Hydrogen Peroxide Quantification

In order to assess the inhibition caused by the production of hydrogen peroxide (H_2_O_2_), antimicrobial assays were performed by adding 5000 Units of bovine liver catalase (Merck, Darmstadt, Germany) in the agar medium.

Kinetics of H_2_O_2_ production was evaluated for *C. inhibens* MIP2551 and compared to *Lactococcus garvieae* CIP 102507^T^. Each strain was grown in Erlenmeyer flasks containing 20 mL of BHI medium, at 26 °C under a 150-rpm shaking. The OD_600 nm_ and H_2_O_2_ concentrations were measured at 0, 3 h, 6 h, 9 h, 15 h, 18 h, and 24 h. H_2_O_2_ concentration was estimated using Dosatest® Peroxide test strips 25, 100, and 1000 (VWR, Fontenay-sous-Bois, France).

### 2.7. Peptidic Activity of Cell-Free Supernatants (CFSs)

*Carnobacterium* spp. cultures were performed in 1 mL of BHI medium in 96 Deepwell plates for 24 h at 26 °C. After centrifugation for 30 min at 5000× *g*, 250 µL of supernatants were transferred into the Multiscreen^TM^ system consisting of a microplate containing 0.22 µm polyvinylidene fluoride (PVDF) filters, Merck Millipore, Burlington, NJ, USA), a 96-well microplate receiver (Greiner Bio-One, Grosseron, France), and a Centrifuge Alignment Device (Merck Millipore, Burlington, NJ, USA). After 5 min of centrifugation at 5000 × *g*, cell free supernatants (CFSs) were collected and stored at −80 °C. 

To determine whether the active compounds were peptidic, 100 µL CFSs were digested by 1 µL of proteinase K (Proteinase K from *Engyodontium album* EC 3.4.21.64; Merck, Darmstadt, Germany) at a final concentration of 0.2 mg/mL for 1 h at 37 °C. The digested CFSs were then spotted on previously inhibited indicator strains seeded in the agar medium, as described above. The absence of inhibition halo after proteinase K treatment evidenced an active antimicrobial peptide. The efficacy of each CFS was compared using *L. monocytogenes* RF191 as the indicator strain. Two-fold serial dilutions of CFS suspensions in BHI were inoculated with 10^6^ CFU/mL of *L. monocytogenes* RF191 and incubated for 24 h at 26 °C. The titration of *Carnobacterium* spp. CFSs was carried out by measuring the growth of *L. monocytogenes* by OD_600 nm_ with a spectrophotometer (Varioskan^TM^, Thermo Fisher Scientific, Waltham, MA, USA). A negative control, CFS of *Carnobacterium maltaromaticum* EBP3034 strain, was included in the analysis. The minimal inhibitory dilution (MID) was determined by the highest dilution of CFS for which no growth of *L. monocytogenes* was measured.

### 2.8. Nucleotide Sequence Accession Number

The Whole Genome Shotgun projects were deposited in DDBJ/ENA/GenBank under the related accession number WNJQ00000000 for the MIP2551 strain, WNJR00000000 for the SF668 strain, and WNJS00000000 for the EBP3019 strain. The version described in this paper is the first one.

## 3. Results

### 3.1. BGC content comparison in *Carnobacterium* species

#### 3.1.1. *Carnobacterium* spp. genome dataset

**Figure 1:**
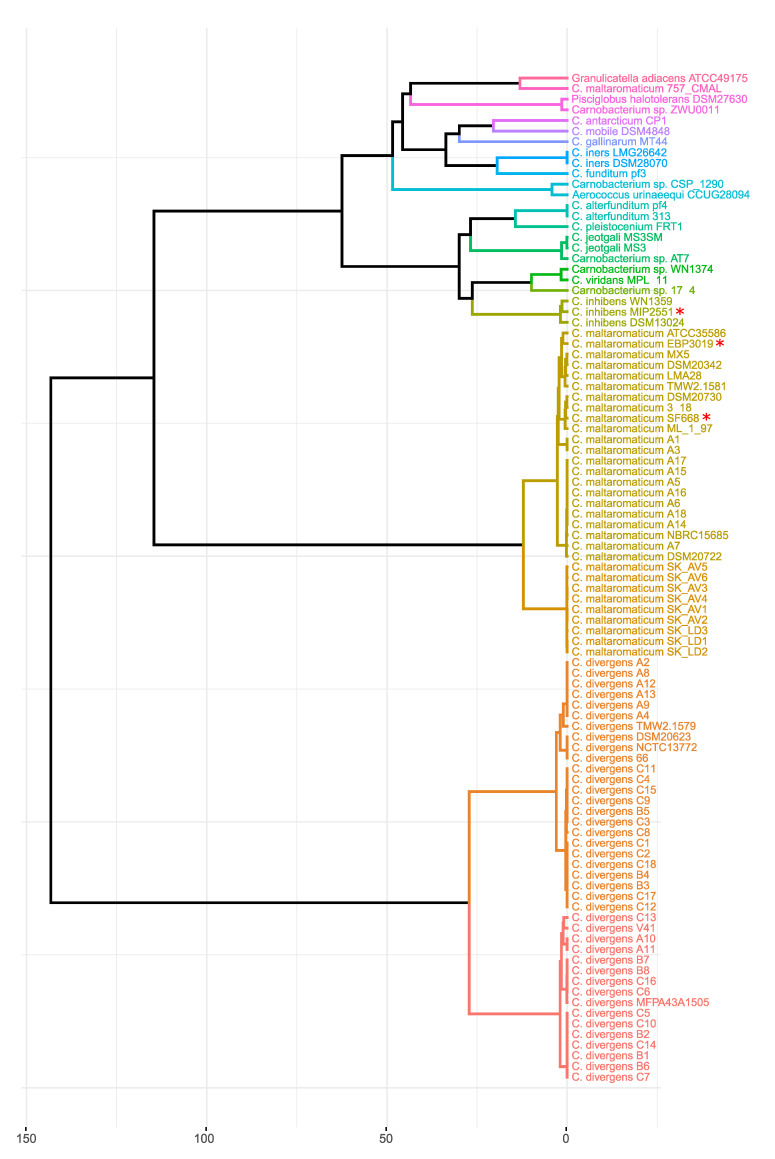
Average Nucleotide Identity (ANI) dendrogram of *Carnobacterium* spp. genomes and closed organisms—*Pisciglobus halotolerans* DSM27630T, *Aerococcus urinaeequi* CCUG28094T, and *Granulicatella adiacens* ATCC49175. The ANI similarity matrix was obtained with OrthoANIu (OrthoANI using USEARCH) provided by EzBioCloud. The dendrogram was constructed with RStudio using hclust {stats} and fviz_dend {factoextra}. The genomes sequenced in this study are highlighted with a red asterisk.

Eighty-nine *Carnobacterium* spp. genome sequences were retrieved from the NCBI database (Table S2). The species affiliation was checked by 16S rDNA-based phylogeny (Figure S1) and by phylogenomics using the ANI (Average Nucleotide Identity) similarity values (Figure 1). Whereas the analysis by 16S rDNA-based phylogeny alone was sufficient for most species’ affiliation, in accordance with the current classification with a high threshold of 99%, the phylogenomics one was required to distinguish the species *C. alterfunditum* and *C. pleistocenium*. The *Carnobacterium* sp. AT7 strain was, thus identified as *C. jeotgali,* and the WN1374 and 17-4 strains as *C. viridans* species. This analysis also detected three erroneously identified *Carnobacterium* sp.—the strain ZWU0011 was identified in this study as *Pisciglobus halotolerans*, the strain 757_CMAL as *Granulicatella adaciens*, and the strain 1290_CSPC as *Aerococcus urinaequii* (Figure 1 and Figure S1). Consequently, these three strains were excluded from our dataset. To simplify further analysis, redundant genomes were also removed. Finally, 39 *Carnobacterium* spp. genomes were selected from the dataset to mine *Carnobacterium* spp. BGCs.

#### 3.1.2. Diversity of Antimicrobial BGCs

**Figure 2:**
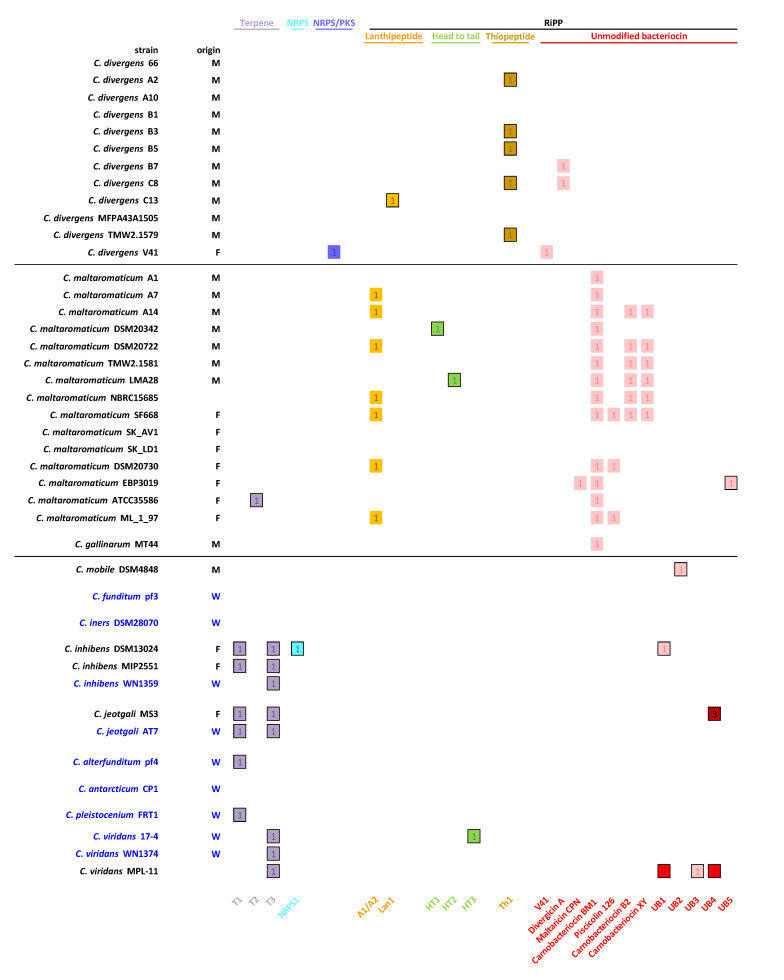
Predicted biosynthetic gene cluster (BGCs) in *Carnobacterium* spp. genomes. Numbers and color intensity indicated the copy numbers of BGCs. Strains from marine origin are colored in blue. Origin: M = meat; F = fish and seafood products; W = water. The three genomes sequenced in this study are highlighted with a red asterisk.

**Figure 3:**
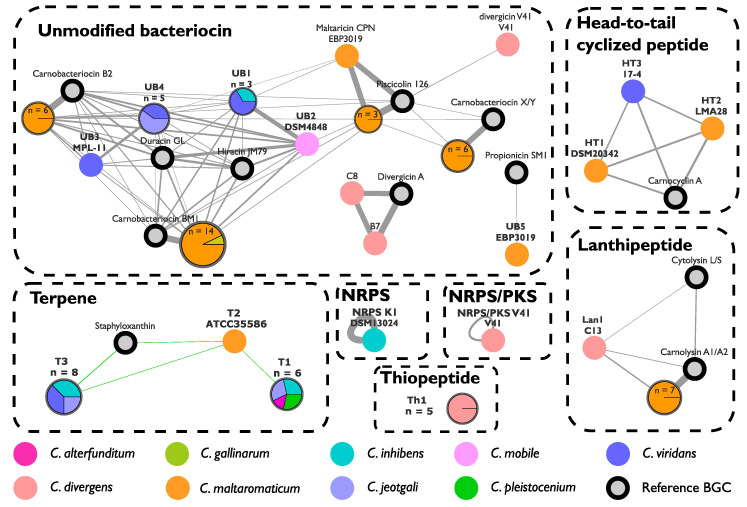
Sequence similarity network of *Carnobacterium* predicted biosynthetic gene cluster (BGCs). The network was generated with BiG-SCAPE, and graphical modifications were done with Cytoscape. A node represents a predicted BGC. For more legibility, several similar BGCs were grouped into a single node. In this case, a pie chart illustrates the represented species, and the size of the node is proportional to the number of BGCs. The thickness of the lines is correlated to the similarity between two nodes. The dotted boxes indicate the different classes of BGCs.

A total of 67 manually annotated BGCs were considered as potentially functional since the required minimal gene set was displayed (Figure 2 and Figure 3). At least one BGC could be predicted in 77% of the analyzed genomes (Figure 2). Twenty-one different BGCs were identified; 14 of them were not described in the literature until now. They were distributed into four classes of natural products (Terpenes, RiPPs, NRPS, NRPS/PKS). *Carnobacterium* spp. strains harbored between zero and six BGCs. Two or three BGC copies were identified in scarce cases (Figure 2). Globally, the BGC content seemed to be species-dependent. The water-sourced strains displayed specialized BGC content, but no significant difference was observed between the food strains isolated from seafood or meat products (Figure 2).

**Figure 4:**
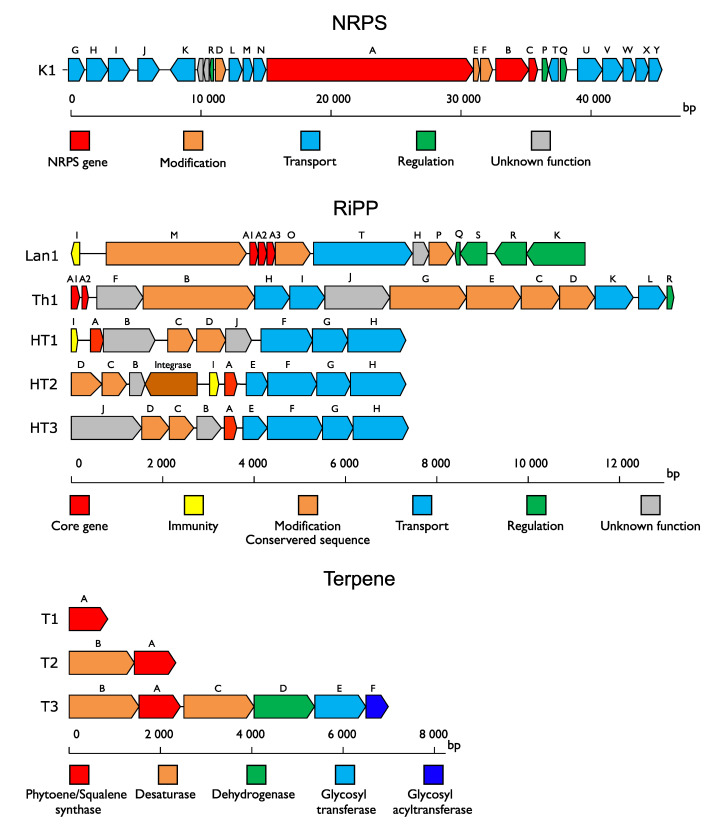
Undescribed biosynthetic gene cluster (BGC) structures. For each undescribed BGC, the gene length, function, and cluster organization are represented and named using letters and numbers when several core genes were predicted in the same cluster.

Terpenes are an important source of natural compounds derived from one or more isoprene unit. A total of 13 terpenes classified into three different groups (T1, T2, and T3) were predicted (Figure 2 and Figure 3). The distribution of these terpenes appeared to be species-related and particularly distributed among environmental strains. Core clusters of T1 and T2 were only composed of, respectively, one and two biosynthetic genes related to terpenes (Figure 4). In both T1 and T2, one gene coding for a putative phytoene/squalene synthase was predicted with a conserved isoprenyl diphosphate synthase domain (*T1A, T2A*). This enzyme is generally involved in the formation of the linear backbone of isoprenoid compounds [49]. *T2B*, an additional gene encoding a bacterial-type phytoene desaturase involved in tetraterpene biosynthesis such as carotenoids, was predicted in the cluster T2 [50]. The T3 BGC was more complex and was composed of six core biosynthetic genes, which code for proteins similar to those in the staphyloxanthin BGC from *Staphylococcus aureus* (Figure 3) with the protein sequence identity varying between 38.3% and 58.9%. Staphyloxanthin is a carotenoid pigment promoting resistance to reactive oxygen species [51].

In addition to the known NRPS/PKS hybrid BGC from *C. divergens* V41, only one NRPS BGC was detected (Figure 2 and Figure 3). The new 43 kb-long NRPS cluster found in the *C. inhibens* DSM13024 strain (also named K1) was composed of an NRPS gene consisting of five complete domain modules (*k1A*) and of a second gene containing the termination module (*k1B*) [52] followed by a phosphopantethienyl transferase (*k1C*) [53] (Figure 4). Genes involved in transport or regulation (*k1G*-*k1Y*), as well as putative auxiliary genes, including a S-adenosyl-L-methionine (SAM)-dependent methyltransferase (*k1D*), an aspartate decarboxylase, and a phosphohydrolase (*k1E* and *k1F*) were also identified.

Two different lanthipeptide BGCs were predicted (Figure 2 and Figure 3). The two-component lanthipeptide carnolysin A1/A2 [54] was detected in approximately half of the *C. maltaromaticum* strains (Figure 2). The amino acid sequence comparison revealed a 100% identity between all predicted canolysin A1/A2 BGCs. A new lanthipeptide BGC, named Lan1, was found in the *C. divergens* C13 strain. The cluster encompassed a *lanM* lanthionine synthetase gene, indicating a class II lanthipeptide [13], followed by three potential precursor peptides (Lan1A1, Lan1A2, and Lan1A3), and a transport system (Figure 4). The best similarity was found with the undescribed sequences from *Bacillus thuringiensis* with an amino acid identity below 68% (Table 1). 

Only one thiopeptide BGC was predicted in a half of the *C. divergens* strains (Th1) (Figure 2). Thiopeptides are RiPPs containing characteristic thiazole rings, described for the first time in *Carnobacterium* genus. The core cluster was composed of two structural genes, named *th1A1* and *th1A2,* with identical sequences (Figure 4 and Table 1). Genes encoding enzymes involved in the structural backbone of the thiopeptide were predicted (*e.g.,* formation of piperidine, dehydropiperidine and pyridine macrocycle, thiazole and thiazoline macrocycle, and Dha and Dhb residues) [13]. They included two LanB-like dehydratases (*th1B* and *th1C*), a potential enzyme involved in cycloaddition (*th1D*), and a cyclodehydratase (*th1G*). The genes involved in the regulation and transport were also predicted, including a putative cyclic autoinducer peptide (*th1R*). Th1 showed a 100% identity with the uncharacterized thiopeptide core sequence of *Enterococcus termitis* (Table 1). 

Only one head to tail cyclized peptide (HT) named carnocyclin A has been reported in *Carnobacterium* species [55], but it was not found in any genome analyzed in this study (Figure 3). Three undescribed HT, namely HT1, HT2, and HT3, with original core sequences were predicted in *C. maltaromaticum* and *C. viridans* (Figure 2 and Table 1). Their molecular mass was estimated between 5.89 and 7.32 kDa. 

Among the predicted RiPPs, 37 out of 52 encoded unmodified bacteriocins (UBs). They were divided into 10 different BGCs. Most of the UBs were identified in *C. maltaromaticum* genomes (Figure 2 and Figure 3). The carnobacteriocin BM1 BGC [56] was found in *the C. gallinarum* MT44 genome and in 85% of the *C. maltaromaticum* genomes. Other described UBs were exclusively found in genomes of *C. maltaromaticum* species—the piscicolin 126 BGC [57] in 15% of the genomes, and the carnobacteriocin B2 and X/Y BGCs [56,58] in 38% of the genomes. The divergicin A BGC [59] was identified in the *C. divergens* B7 and C8 strains and the divercin V41 BGC [48] only in the V41 strain. Four uncharacterized UBs were also predicted in the food-isolated strains *C. inhibens* DSM 13024 (UB1), *C. jeotgali* MS3 (UB4), *C. mobile* DSM 4848 (UB2), and *C. viridans* MPL-11 (UB1, UB3, and UB4) (Figure 2). All four novel genes encoded similar proteins (from 50% to 95% identity to each other) (Figure 3). UB1 and UB2 displayed amino acid similarity with the *Enterococcus durans* duracin GL, whereas UB3 and UB4 with *Enterococcus faecalis* hiracin-JM79 [60,61] (Table 1 and Figure 3).

### 3.2. Antimicrobial Activities of Carnobacterium spp. Isolated from Seafood Products

#### 3.2.1. Inhibition profiles of Carnobacterium spp. strains 

Two hundred sixty *Carnobacterium* spp. strains from seafood products, including 211 *C. maltaromaticum*, 45 *C. divergens*, 3 *C. jeotgali*, 2 *C. inhibens*, 1 *C. funditum*, and 1 *C. viridans* strains were studied. Reference strains *C. alterfunditum* CIP 105796, *C. divergens* CIP 101029, *C. funditum* DSM 5970, *C. gallinarum* NCDO 2766, *C. inhibens* DSM 13024 and WN1359, *C. jeotgali* KCTC 13251, *C. maltaromaticum* CIP 103158, and NCDO 2672, *C. mobile* CIP 103159, *C. pleistocenium* CIP 108033, and *C. viridans* MPL-11 were added to this study. Each *Carnobacterium* species was then represented except for *C. iners* and *C. antarcticum*, which have been recently described [62,63]. The antimicrobial activity profile of these strains was evaluated against 17 indicator bacteria, yeast, and fungi (Table S3).

**Figure 5:**
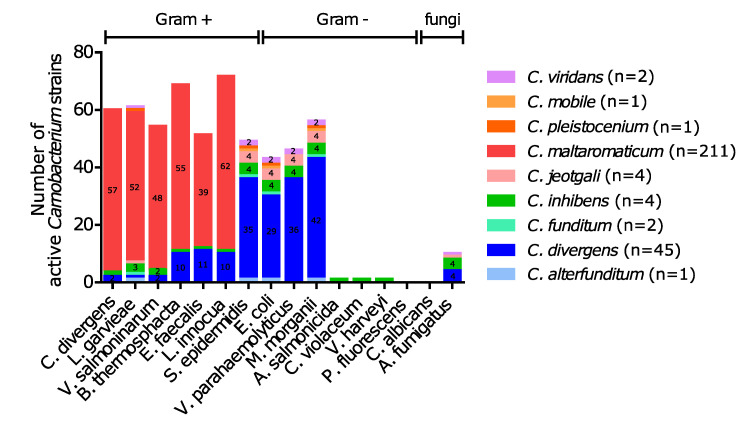
The number of active *Carnobacterium* spp. strains according to the species. *Carnobacterium* spp. strain cultures were spotted on agar plates inoculated with one of the indicator strains. A strain was considered active when the presence of an inhibitory halo could be observed. The number of active strains was indicated inside the bar for species represented by more than one strain. n: total number of *Carnobacterium* spp. strains tested per species.

No inhibition of *Candida albicans* and *Pseudomonas fluorescens* was observed. Approximately 60% of the *Carnobacterium* spp. strains showed antimicrobial activity against at least one of the indicator strains tested. Overall, different inhibition patterns were observed depending on the strain species (Figure 5). *C. maltaromaticum* inhibited all Gram-positive species except *Staphylococcus epidermidis*. In contrast, other *Carnobacterium* species inhibited *Escherichia coli*, *Vibrio parahaemolyticus*, *Morganella morganii*, *Staphylococcus epidermidis*, and *Aspergillus fumigatus.* Interestingly, the *C. inhibens* MIP2551 strain was the sole strain able to inhibit *Aeromonas salmonicida*, *Vibrio harveyi*, and *Chromobacterium violaceum*.

#### 3.2.2. Involvement of H_2_O_2_ Inhibition

**Figure 6:**
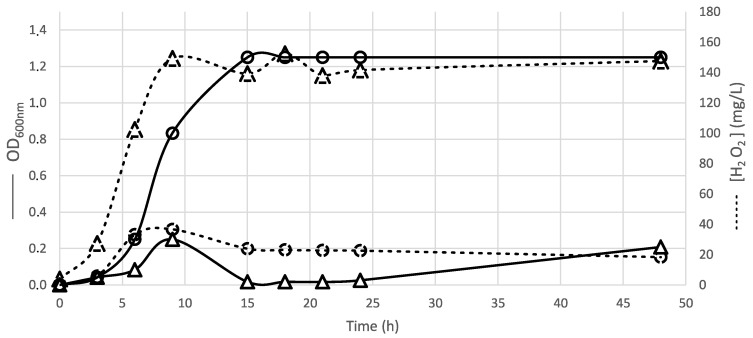
Assessment of H_2_O_2_ production during the growth of *Carnobacteriun inhibens* MIP2551 (triangle) and *Lactococcus garvieae* CIP102507 (circle). Dashed lines: H_2_O_2_ concentration estimated by Dosatest® Peroxide test strips; Solid lines: OD measured at 600 nm. Strains were cultivated at 26 °C, in brain heart infusion (BHI) medium and in shaking conditions.

The addition of catalase into the medium resulted in the loss of the inhibitory effect for 56% of the active *Carnobacterium* spp. strains (Table S3). H_2_O_2_ production is involved in the inhibition mechanism of *S. epidermidis*, *E. coli*, *M. morganii* and *V. parahaemolyticus* by *C. viridans*, *C. mobile*, *C. pleistocenium*, *C. jeotgali*, *C. funditum*, *C. alterfunditum*, *C. inhibens,* and by 67% of *C. divergens* strains. The *C. inhibens* MIP2551 strain was the highest producer of H_2_O_2_; its production time-course was monitored for 24 h and compared to *Lactococcus garvieae*, a species known to produce high inhibitory amounts of H_2_O_2_ (Figure 6). In optimal aeration conditions, the MIP2551 strain began to produce H_2_O_2_ during the exponential phase and reached a maximal concentration of 150 mg/L on the stationary phase, with a maximal OD*_600nm_* of 0.3. This concentration was five times higher than those observed for *Lactococcus garvieae* strain. The same H_2_O_2_ production profile was observed for *C. inhibens* CD344, DSM 13024, and WN1359 strains (data not shown).

#### 3.2.3. Comparison of CFSs Activities

Sixty-five strains (56 *C. maltaromaticum* and nine *C. divergens*) remained active after catalase treatment (Table S3). Their cell-free supernatants (CFSs) were tested against *Lactococcus garvieae* and *L. monocytogenes*. All the 65 CFSs found active against *L. monocytogenes* remained active, suggesting an extracellular active compound. However, only three CFSs remained active against *Lactococcus garvieae*. *C. maltaromaticum* SF668 was the only strain with a CFS active against both bacterial targets. After digestion by proteinase K, all CFSs became inactive, suggesting a peptidic nature for antimicrobial compounds, such as bacteriocins.

**Figure 7:**
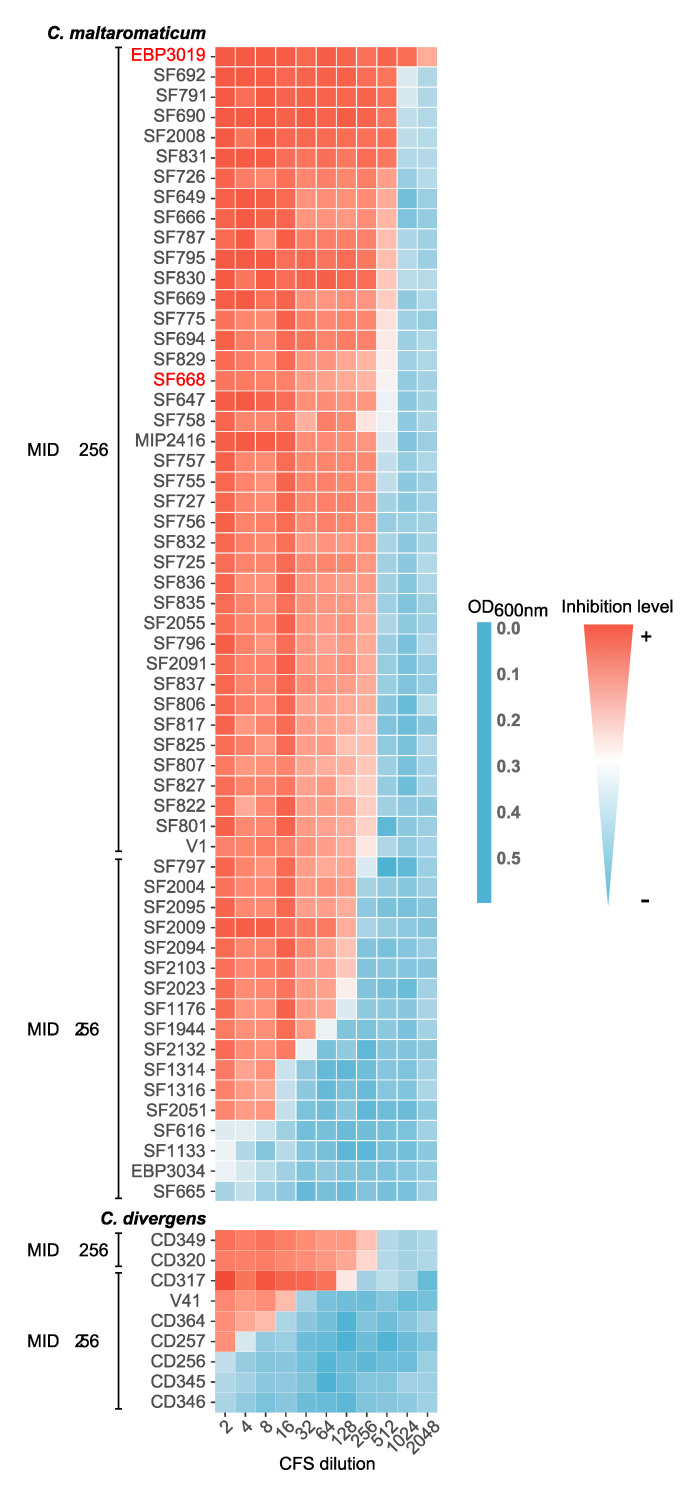
Cell-free supernatant (CFS) efficacy against *Listeria monocytogenes* RF191. The efficacy was evaluated for each strain of *Carnobacterium* spp. by determining their minimal dilution inhibiting the growth of *Listeria monocytogenes* RF191 (MID). The growth of the strain RF191 was measured through OD_600 nm_. EBP3034 strain was used as a negative control. Genomes sequenced in this study are highlighted in red.

CFS efficacy against *L. monocytogenes* was determined and compared for all active strains. The growth of *L. monocytogenes* was measured in CFS-supplemented medium. The 65 active CFSs were diluted to determine the minimal inhibitory dilution (MID) (Figure 7). The MID varied from less than 2 to 256 for *C. divergens*, and less than 2 to 2048 for *C. maltaromaticum* strains. Seventy one percent *C. maltaromaticum* CFSs (40 out of 56) and 22% *C. divergens* CFSs (two out of nine) showed a MID ≥ 256. This result suggested higher activity for *C. maltaromaticum* strains. The *C. divergens* V41, which produces the antilisterial divercin V41 (Table S1) displayed a MID = 16 in the tested conditions. Three *C. divergens* strains, namely CD317, CD320, and CD349, showed higher activity than the *C. divergens* V41 strain. *C. maltaromaticum* V1 strain, which produces the previously described carnobacteriocin BM1 and piscicolin 126 [64] (Table S1), and the EBP3019 and SF668 strains belonged to the most efficient CFS group. A MID greater than 512 was only detected in the EBP3019 strain. This result was confirmed by supplementary triplicate assays allowing the estimation of an average MID of 1024.

### 3.3. MIP2551, EBP3019, and SF668 Genome Specificities

It appeared from all these analyses that *C. maltaromaticum* EBP3019 and SF668 strains displayed interesting antimicrobial activities related to extracellular peptides. SF668 strain was previously shown to have antilisterial effect in cold-smoked salmon and used as bioprotective strain [25]. In addition, *C. inhibens* MIP2551 was shown to produce high amounts of H_2_O_2_. In order to identify potentially original antimicrobial BGCs, the genomes of these three strains were sequenced, assembled, and annotated. Phylogenomic analysis by ANI similarity values for these three strains confirmed the identification using partial 16S rDNA sequencing (Figure 1).

**Figure 8:**
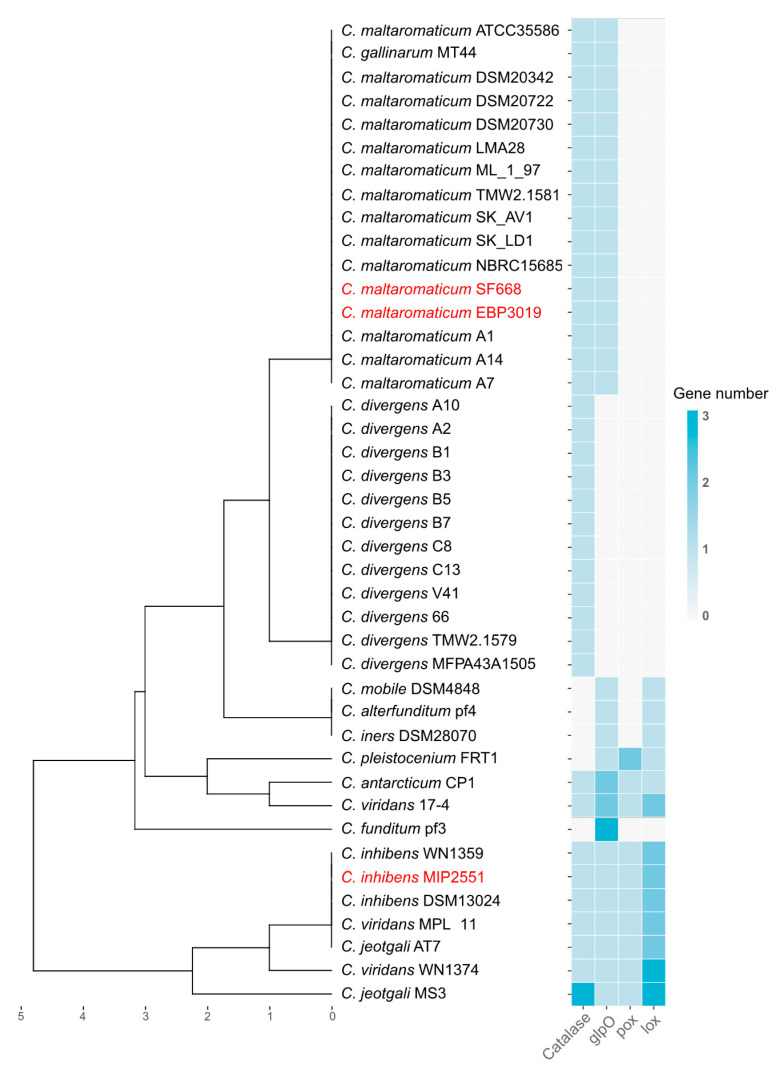
Hierarchical clustering of *Carnobacterium* spp. genomes by the presence of H_2_O_2_ production and decomposition genes: Lactate oxidase (*lox*, EC.1.13.12.4), pyruvate oxidase (*pox*, EC.1.2.3.3), α-glycerophosphate oxidase (*glpO*, EC.1.1.3.21), and catalase (EC.1.11.1.6). Genomes sequenced in this study are highlighted in red.

Genes coding for H_2_O_2_ anabolism and catabolism were identified in the MIP2551 genome and compared to other species (Figure 8). In most cases, it seemed to be species dependent. The catalase gene involved in H_2_O_2_ catabolism was found in MIP2551, and in 88% of the analyzed genomes. Genes coding for the oxidases GplO, Pox, and Lox were found in *C. inhibens* MIP2551 genome. This genetic content was similar to environmental species, which is consistent with H_2_O_2_ inhibition in our bioassay (excepted for *C. antarcticum* not represented). Two to three genes coding for Lox were detected in some genomes as in MIP2551. 

The BGC content of the *C. inhibens* MIP2551, *C. maltaromaticum* EBP3019, and SF668 strains was investigated, and the predicted clusters were dereplicated. The two undescribed terpene BGCs T1 and T3 were predicted in *C. inhibens* MIP2551 genome. A total of five RiPPs were predicted in the SF668 strain, identified as carnolysin A1/A2, piscicolin 126, carnobacteriocin BM1, B2, and X/Y (Figure 2). This equipment corresponded to the largest RIPPs BGC diversity among the *Carnobacterium* spp. genomes. Three RiPPs BGCs were predicted for the *C. maltaromaticum* EBP3019 strain—the highly conserved carnobacteriocin BM1 and the maltaricin CPN BGC [26] along with a new UB named UB5 (Figure 2 and Figure 3). This last BGC displayed the best sequence similarity (49%) with propionicin SM1 from *Propionibacterium jensenii* [65] (Table 1). UB5 is probably extracellular and has a high molecular weight (16.86 kDa).

## 4. Discussion

The use of phylogenomics and, in particular, the analysis of ANI allows new insights into genome evolution and bacterial species definition [66,67]. Within the genus *Carnobacterium*, only the species *C. alterfunditum* and *C. pleistocenium* cannot be distinguished on the basis of 16S rDNA alone. In this study, a combination of 16S rDNA-based phylogeny and ANI similarity ensured accurate strain identification.

The constitution of a reliable *Carnobacterium* spp. genome dataset allowed us to discriminate BGC content depending on species and ecological niches. The number of BGCs, and the class of the predicted molecules appeared to be generally species-specific. For example, carnolysin A1/A2 and carnobacteriocin BM1 are specifically found in *C. maltaromaticum* and thiopeptide Th1 in *C. divergens* strains. The food-sourced species *C. maltaromaticum* appeared to be particularly rich in RiPPs, as well as *C. divergens* to a lesser extent. These observations could be related to their adaptation to animal and food product habitats as previously described [20]. In the same way, RiPPs with original sequences, were identified in strains belonging to the environmental species *C. jeotgali*, *C. inhibens*, and *C. viridans* but isolated in food products (Figure 2). The acquisition of RiPPs in these environmental species could be due to the adaptation to fight against Gram-positive strains largely present in the food microbiome and absent in oceans. This is consistent with a previous study showing that the MIP2551 strain lacking in RiPPs was not competitive in salmon gravlax [68]. Other BGC such as terpene were exclusively predicted in environmental species, with the exception of BGC T2. Terpenes are less studied in bacteria than in plants or fungi, but they do represent a promising source of bioactive compounds. This class of BGCs has not been described yet in the genus *Carnobacterium*. More genome sequences for each species are needed to confirm these observations.

In this study, *C. divergens* and *C. maltaromaticum* isolated from seafood products represented more than 95% of the isolated strains, a ratio which is consistent with that observed in the metagenomic analyses of food [20]. No activity related to the acidification or the production of organic acids was detected in our experiments, which reinforces data from previous studies [69,70]. Genomic analyses revealed the presence of BGCs in most of the *C. maltaromaticum* and *C. divergens* strains while only 60% of the tested isolates were active. Thanks to the reference strains screened for antimicrobial activity, we also observed that some of them showed no activity despite the presence of terpene, NRPS and RiPPs in their genome (e.g., *C. mobile* DSM4848, *C. inhibens* DSM13024, *C. viridans* MPL-11). Since these BGCs remain undescribed, it could be hypothesized that they encode molecules devoid of antimicrobial activity, or active against other strains not tested in this screening. BGCs might also not be expressed under the culture conditions used. Further investigations using culturomic analyses or co-cultures could help to activate such cryptic BGCs [71,72].

The antimicrobial screening showed that environmental *Carnobacterium* species such as *C. inhibens*, *C. jeotgali*, *C. viridans* more likely inhibit bacterial growth through H_2_O_2_ accumulation. High H_2_O_2_ level was in accordance with genomic differentiation of niche species, particularly the presence of *lox* and *pox* genes compared to *C. maltaromaticum*. Most *C. divergens* strains also produced H_2_O_2_ without harboring the known oxidase encoding genes *lox*, *pox*, and *glpO* (Figure 8). *Lox* gene encodes the lactate oxidase responsible for the conversion of lactic acid into pyruvate. It can then be hypothesized that the presence of both *lox* and *pox* genes gives a double advantage to outcompete other microorganisms not only through nutritional competition but also through the production of antimicrobial compounds. *C. inhibens*, *C. jeotgali*, and *C. viridans* species harbor multiple copies of genes encoding Lox probably correlated with H_2_O_2_ production [73], and in parallel the T3 BGC similar to staphyloxanthin-encoding BGC, which has protective properties against H_2_O_2_. A staphyloxanthin-similar function can, therefore, be assumed for the T3 cluster, although no xantho-pigment was produced by the T3 cluster containing strains.

## 5. Conclusions

Combining genome mining, phenotype characterization, and dereplication on a large dataset appeared a relevant approach to avoid rediscovering known active molecules and their BGCs. Thanks to this strategy, new insights into the antimicrobial potential of environmental *Carnobacterium* species suggests that they should be considered for further new biotechnological applications. Furthermore, BGC distribution in *Carnobacterium* spp. showed that the screening of active RiPPs should be performed in food-related strains. Two of them, EBP3019 and SF668, were selected for their activity against *Listeria monocytogenes.* Further studies on EBP3019 supernatant, such as fractionating, purification, mass analysis, and amino acid sequencing have to be conducted to isolate and characterize UB5, whose BGC was not found in any other genome.

## Figures and Tables

**Table 1:** Undescribed ribosomally synthesized and post-translationally modified peptides (RiPPs) sequence prediction. Names were arbitrarily given depending on predicted RiPP classes. Sequence prediction was achieved with AntiSMASH, and the BAGEL leader sequence was predicted with SignalP 5.0. Alignment by Blastp of the core sequence to the non-redundant protein sequence database (nr) was used to predict the RiPP class and the closest known RiPP.

Gene Name	Leader Peptide	Core Sequence	Blastp Prediction	Best Match	Identity	Similarity
Lan1A1	-	MQTTTKSFVGQAFEELSIEEMEVLQGSGDVQPLSSPVSWIATALSAVLCFPGSVS	type 2 lantibiotic	*Bacillus thuringiensis*	48%	72%
Lan1A2	-	MITNQFIGQAFEELSTEEMEVLQGAGEITPYSTIPCAAIISAVWATITKC	type 2 lantibiotic	*Bacillus thuringiensis*	68%	85%
Lan1A3	-	MESKELHQFVGQAFEELSIESMEQLQGSSDISPRTTLPCLESAVVSYEIITMIFCKS	type 2 lantibiotic	*Bacillus thuringiensis*	65%	72%
Th1A1	MEKELSTKDFDLEVELLDLDEVSA	IPETTASSGSTSCSASSTCGSTSCCGSC	thiazolyl-peptide	*Enterococcus termitis*	100%	100%
Th1A2	MERELSVNETTTEDFDLEVELLDSDEVSA	IPETTASSGSTSCSASSTCGSTSCCGSC	thiazolyl-peptide	*Enterococcus termitis*	100%	100%
HT1	MMNVRLTKNYKFYGAISLVLISITIGILFISTTPYIAGA	LGLSTGTATQVVSLISAYQTAAAIVSIVGALTGVGGITSGIVATVLFLLKKQGKAKAALW	circular bacteriocin	*Streptococcus pseudopneumoniae*	67%	80%
HT2	-	MSDLIMEIASSMGISWGVASKVIDLVLAGSSAWAIVAAIVSGGGIIAIGAVAIKALIQSKLKQMGRAAVITW	circular bacteriocin	*Paenibacillus larvae*	49%	70%
HT3	-	MIELTMELMNSMNIGRSTATHVIDLAVAGASAWAIVASIAAGGGIIAIGAVAVRTLIKSKLKKLGYTALVAW	circular bacteriocin	*Paenibacillus larvae*	49%	70%
UB1	MVSGLGLLFSSINVEAATA	YPNGVYCNKTKCWVDWNKAQSEIGKIIVNGWVQSGPWS	duracin GL	*Enterococcus durans*	82%	86%
UB2	MKKNLIKFATVFILVSGLGLLFSSINAEAATA	YPNGVYCNKTKCWVDWNKAQSEIGKIIVNGWIQNGPWS	duracin GL	*Enterococcus durans*	79%	89%
UB3	-	MNGNGVSCTKTKCSVNWGQALTEGTKRWGDNLFGSVSG	hiracin-JM79	*Enterococcus faecalis*	79%	80%
UB4	MGKKILKGLIVSIFLLGIVLFIAPQEAEA	STYYGNGVSCTKKKCSVNWGQSWTEGVQRWGDHLFG	hiracin-JM79	*Enterococcus faecalis*	75%	91%
UB5	MIGEMKMKKNLLFFVVFVLSLSVTPMLASA	ESENKVDMLPDGTTFTFGVPFTTNEFSDDGSYETVTIVSEVTNNTSSSVNIGITPRRIDNGYYIGRAYWINRNGLLSVSIYPNKGASGWTKDRAWDELKRNFSHYANWKNETSLRKQFNCHARPIPPYTGKIPWNLEPSKAATNILTCN	propionicin SM1	*Propionibacterium jensenii* DF1	34%	49%

## Supplementary Materials

The following are available online at https://www.mdpi.com/2076-2607/8/7/1093/s1, Figure S1: 16S rDNA maximum likelihood phylogenic tree, Table S1: Characterized bacteriocins in *Carnobacterium* spp.; Table S2: *Carnobacterium* spp. strains and related genomes used for genome mining; Table S3: Inhibitory activity of *Carnobacterium* cultures and supernatants in the presence or not of catalase.
