# Supplementary material for: New Insight into Antimicrobial Compounds from Food and Marine-sourced Carnobacterium Species through Phenotype and Genome Analyses
Source: Microorganisms. 2020 Jul 21;8(7):1093. doi: 10.3390/microorganisms8071093 (PMC7409045; doi:10.3390/microorganisms8071093)
Supplement: Supplementary file 1 [file microorganisms-08-01093-s001.zip › FigureS1.pdf]

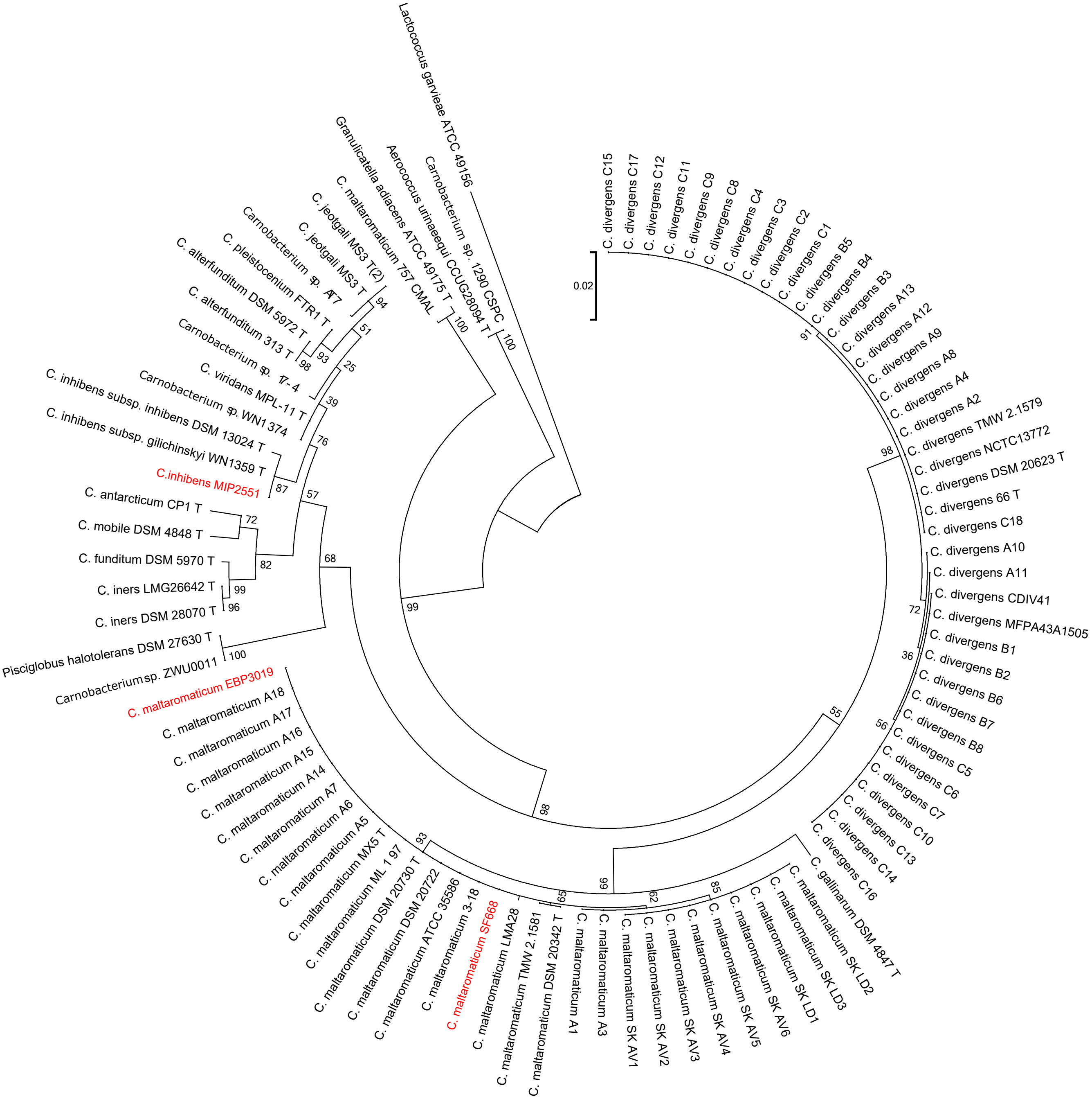

Figure S1. 16S rDNA maximum likehood phylogenic tree. The tree was constructed with MEGA6 using 16S rDNA on *Carnobacterium* genomes and close organisms *Pisciglobus halotolerans* DSM27630T, *Aerococcus urinaeequi* CCUG28094T and *Granulicatella adiacens* ATCC49175. *Lactococcus garvieae* ATCC49156 was used as an outgroup. Genomes sequenced in this study are highlighted in red.
